# Does Vitamin D Have a Role in Diabetes?

**DOI:** 10.7759/cureus.30432

**Published:** 2022-10-18

**Authors:** Tahani M Abugoukh, Afrah Al Sharaby, Abeer O Elshaikh, Malaz Joda, Amna Madni, Ihab Ahmed, Rasha S Abdalla, Kholood Ahmed, Shahd E Elazrag, Nadir Abdelrahman

**Affiliations:** 1 Internal Medicine, Michigan State University College of Human Medicine, East Lansing, USA; 2 Family Medicine - Geriatrics, Michigan State University College of Human Medicine, East Lansing, USA

**Keywords:** type 2 diabetes, type 1 diabetes, vitamin d and diabetes, vitamin d supplementation, vitamin-d deficiency

## Abstract

Vitamin D, a fat-soluble vitamin, acts in the calcium and phosphorus metabolism in its active form (1,25-dihydroxy vitamin D). It may help prevent and treat autoimmune diseases, including diabetes mellitus. Diabetes has become a significant global health issue with a rising incidence and prevalence. A recent focus has been on vitamin D supplementation as part of efforts to discover novel ways to prevent and treat diabetes. Most evidence points to the vitamin D receptors (VDRS) gene in both types of diabetes. The main objective of this study is to analyze how vitamin D affects both type 1 and type 2 diabetes.

In this literature review, we searched the PubMed and Google Scholar databases to collect related articles from 13 papers of different study designs. We found a significant association between vitamin D deficiency and type 1 and type 2 diabetes development. It has been shown that vitamin D supplements have a promising effect in reducing glycated hemoglobin (HbA1c) in patients with type 1 diabetes, with no significant impact on the incidence or improvement of type 2 diabetes. Patients with diabetes and people at high risk of diabetes need the appropriate amount of vitamin D; therefore, regular testing and vitamin D supplementation are advised for the management and prevention of diabetes. Additional randomized studies with bigger sample sizes and longer-term trials are required to fully explore the benefits of vitamin D supplementation in patients with type 1 and type 2 diabetes.

## Introduction and background

**Figure 1:**
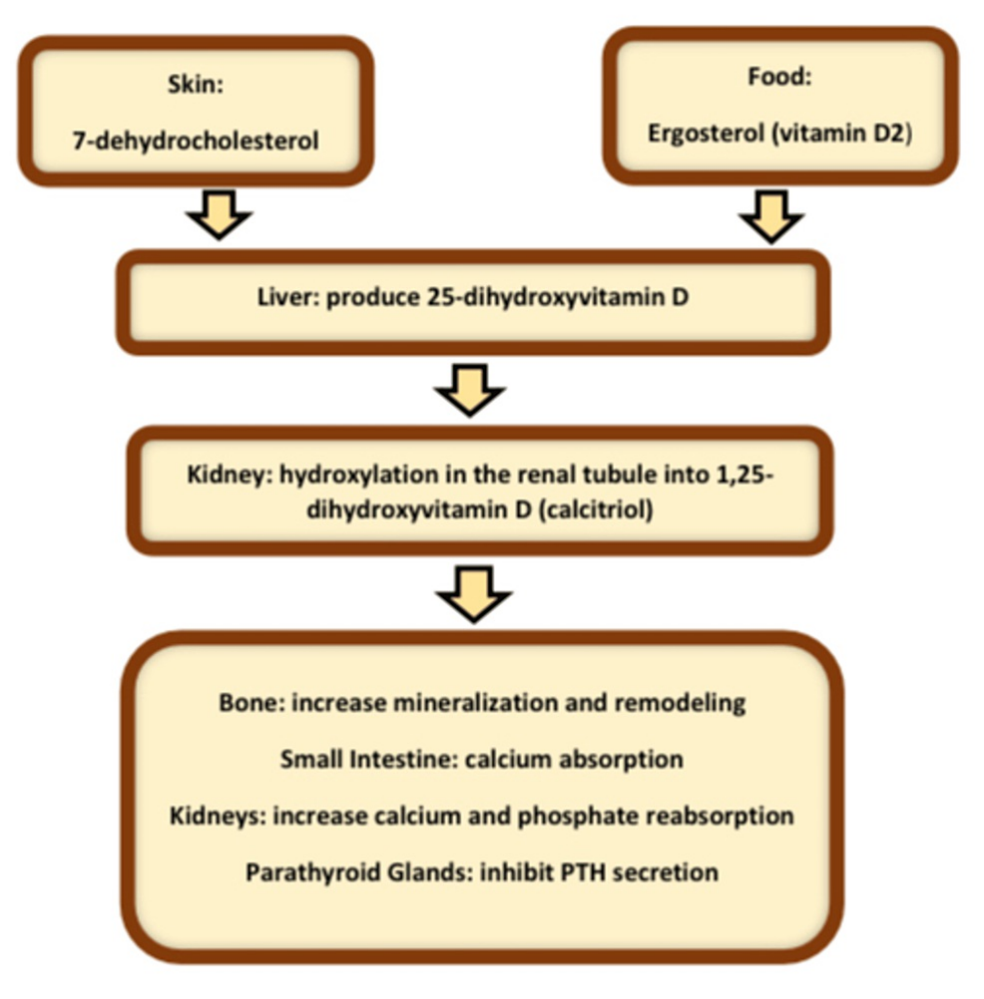
Vitamin D metabolism PTH = parathyroid hormone Figure courtesy: Dr. Ihab Ahmed

Vitamin D is a class of fat-soluble steroid hormones. It has an established role in calcium homeostasis and bone health [1,2]. It is found in fish liver oil, fatty fish, mushrooms, milk, cereals, egg yolks, liver, and vitamin D supplements [1,3]. It can also be synthesized in the body when it is exposed to sunshine [1]. Vitamin D3 (cholecalciferol) and vitamin D2 (ergocalciferol) are the two physiologically active forms [1]. In its dietary, supplemented, or cutaneous synthesized form, vitamin D (Vit D) is delivered to the adipose tissues for storage or to the liver for activation to 25-hydroxyvitamin D [25(OH)D], the major circulating form [2]. This form is then converted to 1,25-dihydroxyvitamin D in the kidneys, the systemically circulating active form that binds to vitamin D receptors (a transcription factor) in different tissues [2], as seen in Figure 1.

The 1,25-dihydroxy vitamin D aids in the metabolism of calcium and phosphorus as well as intestinal calcium absorption, ensuring appropriate levels of these minerals for physiological activities and bone mineralization. It also promotes bone health by keeping parathyroid hormone (PTH) levels at a physiologic range, reducing fall risk and fractures [2]. In addition, it dramatically impacts the proper function of the musculoskeletal, immune, nervous, and cardiovascular systems. Vitamin D has recently been suggested to help prevent and treat autoimmune diseases such as type 1 diabetes mellitus, multiple sclerosis, SLE, or rheumatoid arthritis [4,5]. Vitamin D deficiency increases the risk of rickets and osteoporosis and is linked to various disorders [6].

Diabetes mellitus (DM) is a metabolic disorder characterized by abnormally high blood glucose levels [7]. Diabetes mellitus is becoming a worldwide health issue [8], and the global prevalence of diabetes is expected to rise by 10.1% by 2030 [9]. It is responsible for about 1 million deaths per year, making it the ninth most prominent cause of death [8]. The rising incidence necessitates targeted screening for diabetes and prediabetes in high-risk groups to avoid the disease's emergence and progression [10]. There are many types of diabetes, including type 1, type 2, maturity-onset diabetes of the young (MODY), gestational diabetes, neonatal diabetes, and secondary causes related to endocrinopathies and steroid usage [6]. The most common types and that we will focus on in this study are type 2 diabetes, which accounts for more than 90%-95% of diabetes is caused by a combination of insulin resistance and an insufficient compensatory insulin production response, and type 1 diabetes, which accounts for around 5%-10% of all diabetes cases is caused by an absolute lack of insulin secretion [11-13].

In both experimental and epidemiological research studies, vitamin D insufficiency has been linked to lower insulin release, insulin resistance, and type 2 diabetes [14]. In this literature review, we aim to explore how vitamin D affects patients with type 1 and type 2 diabetes. A review of the current literature was conducted via PubMed and Google Scholar using the search term Vit D deficiency/ supplementation and type 1 and type 2 Diabetes. The search was limited to studies published in English from 2008 to 2022, including systematic review, literature review, randomized controlled trials, and meta-analysis. Two independent reviewers reviewed the studies, and the rest of the team verified the results to avoid bias. We excluded animal studies and other types of diabetes.

## Review

Association between vitamin D and diabetes

Marino and Misra conducted a review study emphasizing meta-analyses and randomized controlled trials. They mentioned that Vit D receptors (VDRs) are found in pancreatic beta cells, which also express 1-hydroxylase (encoded by CYP27B1), and the human insulin gene promoter contains a vitamin D response element [15]. In addition, vitamin D regulates T-cell responses and may protect beta cells from immune attack [15].

Recently, Infante and his team, in their review study, mentioned that inflammation has a significant role in the pathogenesis of type 1 diabetes (T1D) via cytokine and chemokine production by both beta cells and immune cells, which lead to beta cell dysfunction and apoptosis. Calcitriol and its analogs have been shown to prevent IL-1-induced inhibition of beta-cell function and IFN-y-stimulated beta-cell expression of MHC class I and class II molecules [16]. They also demonstrated the link of VDR gene polymorphisms to T1D [16].

Palomer et al. reviewed and identified data that supports VDR as a potential gene for type 2 diabetes (T2D) susceptibility. They found that at least four different genetic factors or VDR modifications may have a role in the development of type 2 diabetes mellitus: alteration of calcium metabolism, modification of adipocyte activity, modification of insulin secretion, and modification of cytokine production [17].

Lips et al., in their review, showed that low vitamin D is associated with T2D, which attributed this to two main causes; first, vitamin D stimulates insulin secretion by pancreatic b cells; thus, vitamin D deficiency is associated with insulin resistance [14]. Second, vitamin D deficiency causes inflammation and increases inflammatory markers. Moreover, it's associated with metabolic syndrome development. In addition, genetic polymorphism of vitamin D may lead to impaired glycemic control [14].

Wang et al. conducted a case-control study of 2659 Chinese participants. They explained that the association between low serum levels of vitamin D and T2D is due to altering the concentration of total cholesterol, low-density lipoprotein cholesterol, and high-density lipoprotein and may lead to impaired fasting glucose and type 2 diabetes [18].

In conclusion, there are different suggested mechanisms of association between vitamin D and the development of type 1 and type 2 diabetes.

Effect of vitamin D deficiency on diabetes

Infante et al., in their review study, concluded that hypovitaminosis D is very prevalent in children with type 1 DM (T1D) [16]. It is an essential environmental factor suggested in the development of T1D and may have a role in the pathogenesis and determining the risk of development in the first years of life, especially in children with high genetic risk [16]. Therefore, it should be diagnosed and treated early [16].

Moreover, Marino and Misra supported Infante's idea in their review study. They suggested that low vitamin D has a role in the development of type1 DM [15].

Additionally, Aljabri and Bokhari, in their nonrandomized control trial on 80 participants with type 1 diabetes, supported this idea and raised the point that low vitamin D is associated with insulin resistance and beta cell death, contributing to the development of T1D [19].

In contrast, Najjar et al. conducted a systematic review and meta-analysis of 10 studies [20]. They concluded that there is no prominent effect of genetically determined reduction of 25(OH)D concentrations by selected polymorphisms on T1D risk [20]. In their review study, Lips et al. concluded that vitamin D deficiency is associated with the development of type 2 DM [14]. Wang et al., in their case-control study, found an inverse relationship between serum levels of 25(OH)D3,25(OH)D2 and a total of 25(OH)D and impaired fasting glucose; they explained this negative relationship to be due to altering lipid metabolism [18].

Furthermore, when Zheng et al. conducted a meta-analysis of 120,618 participants of European descent, they concluded that, although there was a significant inverse observational connection between 25(OH)D and T2D, Mendelian randomization (MR) analysis revealed a lack of support for a causal relationship between 25(OH)D and T2D [21].

The studies mentioned above showed a significant relationship between vitamin D deficiency and the development of both type 1 and type 2 diabetes, except for one study, which reported no relation between vitamin D deficiency and T1D.

Role of vitamin D supplementation on diabetes

Li et al. conducted a systematic review and meta-analysis; 20 randomized controlled trials comprising 2703 participants were included in their review [22]. Vitamin D supplementation successfully enhanced serum 25(OH)D and improved insulin resistance [22]. This effect was notably noticeable when vitamin D was administered in high doses over a short period of time to non-obese individuals who were vitamin D deficient, of Middle Eastern origin, or had good glycemic control at the start [22]. 

In contrast, Jennifer et al. systematically reviewed 35 randomized control trials (RCTs) with 43,407 patients on the effects of vitamin D supplementation on glycaemic control or diabetes prevention [23]. They found that supplementation with vitamin D does not appear to enhance glycaemic control or insulin resistance in the short term [23].

Similarly, Pittas et al. conducted an RCT where 2423 individuals were randomly assigned [24]. Vitamin D3 supplementation at a dose of 4000 IU per day did not result in a significantly lower risk of diabetes than a placebo in those at high risk of type 2 diabetes who were not selected for vitamin D deficiency [24]. Zheng et al., in their meta-analysis study, also did not support the use of Vit D supplements for T2 D prevention [21].

Additionally, Zhao et al. conducted a meta-analysis of 4 prospective cohort studies with 187592 participants and 9456 incident cases. They found no link between total vitamin D intake and the incidence of type 2 diabetes [25].

Aljabri and Bokhari, in their nonrandomized, nonblinded clinical trial on 80 participants with type 1 diabetes whose vitamin D levels were below 50 nmol/L, were given 4000 IU of vitamin D3 and Calcium supplements [19]. The levels of HB A1C and 25-hydroxyvitamin D were tested at the start and after 12 weeks. They found that vitamin D supplementation improved glycemic control in type 1 diabetes mellitus patients [19].

Similarly, Mohammadian et al. conducted a systematic review and meta-analysis. They looked at 44 patients with type 1 diabetes who were younger than 17 years old and were given a vitamin D supplement. They concluded that in children with type 1 diabetes, HbA1C improves with vitamin D3 supplementation in all glycemic control groups, including HbA1C less than 7.8, 7.8-9.9, and >9.9. This supplementation helped the group achieve better glycemic control (p-value 0.0001) [26].

In contrast, Infante et al. mentioned in their review that the ability of vitamin D to stop or reverse islet autoimmunity prompted researchers to look into vitamin D supplementation as adjuvant immunomodulatory therapy for the treatment of T1D [16]. Various vitamin D dosages, formulations, and analogs have been examined in multiple studies. However, there is still a lack of evidence on vitamin D supplementation and beta-cell function preservation in T1D [16].

As mentioned above, most of the included studies on type 2 diabetes concluded that there is no effect of supplementation on the incidence or improvement of type 2 diabetes, except one study revealed an improvement of insulin resistance after Vit D supplementation. While supplementation of Vit D was significantly effective in the improvement of type 1 diabetes in most studies, one study showed no evidence of that improvement.

The characteristics of included studies are summarized in Table 1.

**Table 1:** Summary of the characteristics of included studies T1D = type 1 diabetes, T2D = type 2 diabetes

Author	Type of Study	Participants	Findings
Lips et al. [14]	Review		Low vitamin D is associated with insulin resistance and type 2 diabetes.
Marino and Misra [15]	Review		Low vitamin D has a role in the development of Type1 DM, suggested by the presence of vitamin D response element in the human insulin gene promoter. In addition, vitamin D regulates T-cell responses and may protect beta cells from immune attacks.
Infante et al. [16]	Review		Hypovitaminosis D is an important environmental factor in the development of T1D and may have a role in the pathogenesis and determining the risk of development in the first years of life. However, evidence on vitamin D supplementation and beta-cell function preservation in T1D is still lacking.
Wang et al. [18]	case-control study	2659 Chinese participants	They found that low serum levels of 25(OH)D3,25(OH)D2 were associated with impaired fasting glucose in patients with T2DM; they explained this negative relationship to be due to altering lipid metabolism and altering the concentration of total cholesterol, low-density lipoprotein cholesterol high-density lipoprotein which leads to impaired fasting glucose and type 2 DM.
Aljabri and Bokhari [19]	clinical trial	Eighty participants with T1D whose vitamin D levels were below 50 nmol/L	Low vitamin D is associated with insulin resistance and beta cell death, contributing to the development of T1D. Additionally, Vitamin D supplementation improved glycemic control in T1D patients.
Najjar et al. [20]	systematic review and meta-analysis, including10 studies		No major effect of genetically determined reduction of 25(OH)D concentrations by selected polymorphisms on T1D risk.
Zheng et al. [21]	meta-analysis	120618 participants of European descent	MR analysis revealed a lack of evidence for a causal link between 25(OH)D and T2D despite a high inverse observational association between the two diseases. The result was against the use of Vit D supplements to prevent T2D.
Li et al. [22]	systematic review and meta-analysis, including 20 RCTs	2703 participants.	Vitamin D supplementation successfully enhanced serum 25(OH)D and improved insulin resistance.
Jennifer et al. [23]	A systematic review, including 35 (RCTs)	43407 diabetic patients	They found that supplementation with vitamin D does not appear to enhance glycemic control or insulin resistance in the short term.
Pittas et al. [24]	Randomized control trial	2423	In people at high risk of type 2 diabetes who were not specifically chosen for vitamin D deficiency, vitamin D3 treatment at a level of 4000 IU per day did not result in a significantly reduced risk of diabetes than placebo.
Zhao et al. [25]	A meta-analysis, including four prospective cohort studies	187592 participants and 9456 incident cases	They found no connection between total vitamin D intake and the incidence of type 2 diabetes.
Mohammadian et al. [26]	Systematic review and meta-analysis	44 patients with T1D who were younger than 17 years old	HbA1C improves with vitamin D3 supplementation in all glycemic control groups in children with T1D and vitamin D insufficiency.

Limitations

Although this review contains several randomized control trials and meta-analyses, it still has certain limitations. In this review, we only looked at publications published in English between 2008 and 2022 that are available for free. Additionally, some studies have a small sample size with short-term trials.

## Conclusions

Although the function of vitamin D in regulating blood glucose is still not fully understood, vitamin D status appears to play a role in the onset and management of diabetes mellitus. To understand how vitamin D affects type 1 and type 2 diabetes, we collected several studies that showed a relation between vitamin D deficiency and the onset of both types of diabetes. Most studies conducted to discuss the effects of vitamin D on glucose metabolism supported the hypothesis that appropriate vitamin D supplementation may improve the metabolic regulation of glucose levels in type 1 diabetes; in contrast, most of the studies showed no significant improvement in the levels of hemoglobin A1C in type 2 diabetes with supplementation of vitamin D. As the prevalence of diabetes rises and vitamin D insufficiency is quite common, more investigations and research are required to figure out the exact link between vitamin D and diabetes.
